# A Length-Adaptive LLM-Enhanced Method for Drug–Target Interaction Prediction

**DOI:** 10.3390/ijms27146212

**Published:** 2026-07-12

**Authors:** Hengli Zhao, Yongyi Zhang, Xinyu Tian, Yilin Chen, Wei Song

**Affiliations:** 1 School of Computer Science and Artificial Intelligence, Zhengzhou University, No. 100, Science Avenue, Zhengzhou 450001, China; hengli0650@stu.zzu.edu.cn (H.Z.); zyy_yongyi@stu.zzu.edu.cn (Y.Z.); xinyutian@gs.zzu.edu.cn (X.T.); ylchen@stu.zzu.edu.cn (Y.C.); 2 National Supercomputing Center in Zhengzhou, Zhengzhou 450001, China

**Keywords:** drug–target interaction, length-adaptive fusion, large language model, multi-head attention, deep learning

## Abstract

Drug–target interaction (DTI) prediction is central to drug repurposing. Although existing methods integrate multi-dimensional features and have achieved certain progress, they rely heavily on molecular structure or graph structure information. This makes it difficult to capture complex biomedical semantic associations, leading to insufficient semantic information. To address this issue, this paper proposes BioLA-DTI, a large language model (LLM)-enhanced method for drug–target interaction prediction. The method leverages LLMs to provide semantic information for drugs and proteins. Furthermore, existing methods apply uniform weighting strategies to proteins with significantly different lengths. This introduces noise for short proteins and under-represents long ones. To tackle the feature imbalance problem caused by protein-length variation, this work designs a length-adaptive fusion module. This module dynamically weights the LLM-derived semantic features against structural features based on the actual protein-sequence length. Experiments on two benchmark datasets show that BioLA-DTI outperforms existing baseline methods across multiple evaluation metrics, particularly in cold-start scenarios. Case studies demonstrate that this model can provide valuable references for drug discovery. Ablation studies further confirm the critical roles of LLM enhancement and length adaptation modules.

## 1. Introduction

Drug discovery is a lengthy, costly, and high-risk process [1]. The identification of drug–target interactions is a core bottleneck in this process. Against this background, computational drug–target interaction (DTI) prediction methods have emerged and gained increasing attention. These methods replace certain wet-lab experiments with in silico simulations. They can rapidly screen potential candidate molecules in the early stages of drug discovery. This effectively controls the cost and scale of subsequent experiments [2].

In recent years, deep learning technology has been widely applied in the field of drug discovery. Particularly in drug–target interaction (DTI) prediction, deep neural network-based methods have significantly improved the efficiency of drug repositioning [3,4,5]. In addition, approaches based on heterogeneous network integration provide complementary strategies for DTI prediction [6]. DTI prediction generally requires the integration of multi-source information, including molecular drug sequences, molecular graph structures, and protein amino acid sequences. Traditional methods rely on manual feature engineering. In contrast, deep learning models can automatically extract hierarchical representations from raw data. This advantage has made them the current mainstream technical approaches [7,8].

Deep learning has achieved substantial progress in DTI prediction [9]. However, most existing models adopt vector concatenation or simple summation. They use these operations to fuse multi-modal features of drugs and proteins [10,11]. Such linear fusion strategies struggle to capture the nonlinear correspondence between local chemical substructures of drugs and functional regions of proteins. This limitation hinders the model’s ability to learn complex binding patterns [12,13].

**Figure 1:**
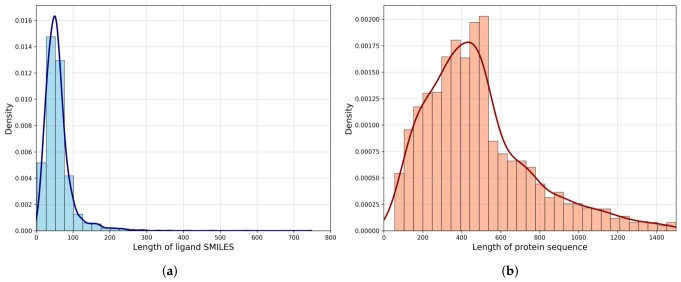
Distributions of ligand SMILES lengths and protein sequence lengths in the BioSNAP dataset. (**a**) Density distribution of ligand SMILES lengths. (**b**) Density distribution of protein sequence lengths.

**Figure 2:**
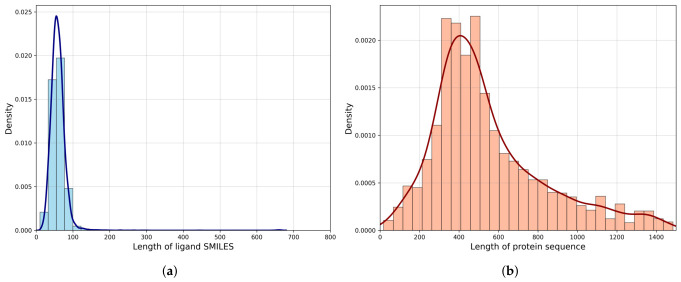
Distributions of ligand SMILES lengths and protein sequence lengths in the BindingDB dataset. (**a**) Density distribution of ligand SMILES lengths. (**b**) Density distribution of protein sequence lengths.

Meanwhile, protein sequences exhibit inherent length heterogeneity [14]. Protein length is not merely a measure of molecular size. It is a key physical parameter that determines interaction patterns, functional space, and regulatory complexity [15,16]. In drug design, systematic analysis of protein length helps distinguish differential inhibition strategies between normal and aberrant pathways. This offers a new avenue for improving intervention precision [15]. Protein-length heterogeneity requires predictive models to possess multi-scale representation capabilities. Ignoring this information may compromise the effective capture of cooperative relationships [16]. Therefore, adopting protein length as the physical basis for subsequent weighting and analysis is biologically and computationally well justified. To reconcile data-driven threshold selection with prior knowledge from the literature, the length distribution of proteins in the experimental dataset was first analyzed, as shown in Figure 1b and Figure 2b. Inflection points were observed at approximately 300 and 800 residues. Previous studies have indicated that 300 residues serve as an important statistical boundary for distinguishing short from long proteins in translational regulation [17]. The 800-residue threshold corresponds to the upper limit of the domain-size distribution; proteins exceeding this length often consist of multiple tandem domains [18]. Based on these findings, 300 and 800 residues were adopted as the cutoffs for classifying short and long proteins. Specifically, short proteins (e.g., fewer than 300 residues) typically consist of a single domain or a few compactly arranged domains, with functional sites (e.g., binding pockets and catalytic centers) concentrated along the sequence. For such proteins, local sequence motifs and co-evolutionary signals are often sufficient to encode the key features required for function [19]. In contrast, long proteins (e.g., more than 800 residues) are often composed of multiple domains in tandem, with key residues sparsely distributed among large, non-functional regions. Relying solely on raw sequences makes it difficult to capture long-range residue cooperativity and cross-domain functional coupling [20,21]. Consequently, long proteins require supplementary prior knowledge, such as function-enriched descriptions generated by large language models [22].

Current methods apply uniform feature processing and fusion pipelines to proteins of all lengths. For short proteins, this introduces irrelevant information and causes performance degradation. For long proteins, the lack of external knowledge leads to insufficient representation. The pre-training of large language models (LLMs) on biomedical texts provides new opportunities to introduce external prior knowledge [23]. Generative models such as BioGPT [24] can produce protein descriptions with rich biomedical semantics. Encoder models such as PubMedBERT [25] can provide high-quality semantic representations for protein-related biomedical texts. Both types of models hold promise in terms of filling the knowledge gaps of traditional sequence features. To date, few studies have reported the dynamic adjustment of the fusion weights between external LLM knowledge and underlying sequence or structural features according to protein length.

To solve these problems systematically, BioLA-DTI, a length-adaptive, LLM-enhanced method, is proposed. As shown in the protein-length density distributions of BindingDB and BioSNAP (Figure 1b and Figure 2b), protein sequences follow a long-tailed distribution with obvious length differences. Most proteins have 200–600 residues, while many are shorter than 300 or longer than 800 residues. Based on this distribution, the model dynamically adjusts fusion weights between semantic and structural LLM features according to protein length. Proteins are divided into three groups: <300, 300–800, and >800 residues, with learnable parameters of $\omega_{1},\omega_{2}$, and $\omega_{3}$ for each group. This grouping reflects biological heterogeneity in protein-domain organization and functional site density. Short proteins rely on their own sequence–structural features to avoid noise. Long proteins utilize LLM prior knowledge to compensate for sparse intrinsic information. Moreover, a gating mechanism is adopted for feature enhancement, and learnable multi-head attention is adopted for deep cross-modal drug–protein feature interaction.

A semantically enhanced representation method with length-adaptive fusion is proposed. Comprehensive advantages and strong cold-start performance are validated via the BioSNAP and BindingDB datasets.

## 2. Results

### 2.1. Baseline

To evaluate the performance of the proposed method, it is compared with several state-of-the-art baseline models on both the BioSNAP and BindingDB datasets. The compared baseline models include DeepConv-DTI [26], MolTrans [3], TransformerCPI [27], GraphDTI [28], MCANet [29], DrugBAN [30], MGNDTI [31], and MGMA-DTI [32].

### 2.2. Analysis of Performance

**Figure 3:**
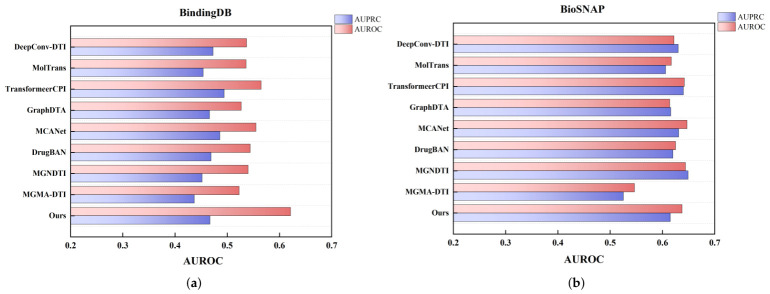
Performance comparison under cold-start scenarios. (**a**) Results on BindingDB. (**b**) Results on BioSNAP.

Table 1 presents the performance of all methods on the BioSNAP and BindingDB datasets, where bold numbers denote the optimal results. On the BindingDB dataset, the model achieves the best results in AUROC, recall and F1 score, demonstrating superior prediction accuracy and classification reliability. Although the AUPRC and accuracy of the model are slightly lower than those of MGMA-DTI [32], the performance gap is negligible. More importantly, the cold-start experiment better reflects practical application value and generalization ability (as shown in Figure 3). In this setting, the model significantly outperforms MGMA-DTI [32]. These results verify the effectiveness and practicability of the proposed strategy.

On the BioSNAP dataset, the method outperforms all baseline models across four key evaluation metrics. In particular, the AUROC and AUPRC are improved by more than 1.5 percentage points, showing stronger overall discrimination and prediction capability. Despite a slight gap in the F1 score compared with MGMA-DTI [32], the substantial improvement in comprehensive performance and core evaluation metrics fully demonstrates the superiority of this method.

It should be noted that the performance differences among baseline methods reflect their distinct design priorities. Specifically, MGMA-DTI [32] achieves marginal improvements in AUPRC and accuracy on the BindingDB dataset. This advantage is attributed to its specially designed graph aggregation module, which effectively extracts local substructure features from molecular drug graphs. However, this model does not incorporate pre-trained language models for semantic protein enhancement and lacks external prior semantic knowledge. Consequently, its generalization capability is constrained under cold-start scenarios.

DeepConv-DTI [26] employs local convolution and global max pooling to capture protein binding-site features. MolTrans [3] explicitly models fine-grained associations between drug substructures and protein subsequences via a Co-Attention mechanism, achieving significantly deeper interaction modeling than conventional methods. TransformerCPI [27] captures inter-sequence dependencies based on the Transformer decoder. Although these three methods achieve reasonable benchmark performance across various datasets, none of them introduces pre-trained semantic knowledge. Therefore, their performance upper bound is limited in scenarios where semantic information is scarce.

DrugBAN [30] introduces a domain adaptation mechanism, while MGNDTI [31] adopts a multi-modal gated fusion mechanism. Both methods achieve competitive results across multiple metrics, confirming the beneficial impact of multi-modal fusion on DTI prediction. However, neither of them incorporates pre-trained language models, nor do they explicitly design adaptive fusion strategies targeting protein sequence-length heterogeneity.

The above analysis indicates that existing methods have room for improvement in terms of both semantic enhancement and length adaptation, which are exactly the core motivations of the proposed method. Combining the results on both datasets, the proposed method introduces LLM semantic enhancement with length-adaptive fusion, which can more effectively capture the deep interaction patterns between drugs and targets and exhibits prominent overall superiority.

### 2.3. Ablation Studies

#### 2.3.1. General Ablation Experiments

Ablation experiments are conducted on the BioSNAP dataset by gradually removing core modules. M1 denotes the model without the gating module, M2 represents the model removing the enhanced LLM component, M3 refers to the model that replaces the multi-head attention in the feature fusion module with direct concatenation, and Full stands for the complete proposed model.

**Figure 4:**
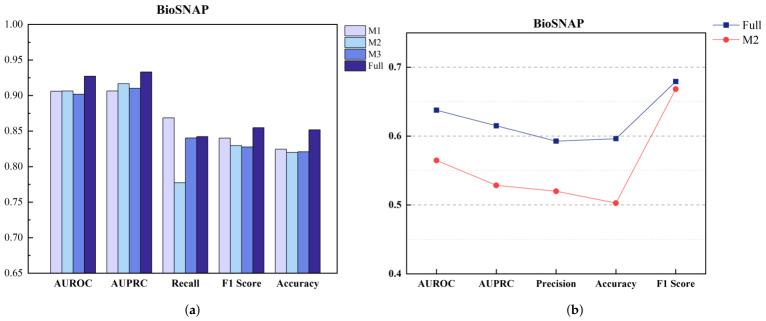
Experimental results on the BioSNAP dataset. (**a**) Experimental ablation results. (**b**) Comparison of metrics in cold-start settings.

AUROC, AUPRC, recall, F1 score and accuracy are adopted as evaluation metrics, and the experimental results are illustrated in Figure 4a.

The ablation results are analyzed as follows:

When Module 1 (M1) is removed, the overall model performance declines slightly, but there remains a clear gap relative to the Full model in core metrics such as AUROC and AUPRC. This indicates that the gating mechanism can effectively enhance feature representation capability and provides steady performance gains for the model.

When Module 2 (M2) is removed, the recall drops most significantly by approximately 0.06 compared with the Full model, accompanied by a noticeable decline in AUPRC, the F1 score and other metrics. This demonstrates that the biomedical semantic enhancement module built based on BioGPT and PubMedBERT is critical for the model’s ability to identify positive samples and serves as a key factor in improving the sensitivity of interaction prediction.

Model 3 (M3) is set as an ablation group. The cross-modal multi-head attention fusion module is removed from the full model to evaluate its individual contribution. After removing M3, all model metrics drop moderately. This proves the module can capture implicit drug–protein interactions and improve prediction performance. The performance drop as a result of removing M3 is slighter than that resulting from the removal of the LLM semantic enhancement module. Hence, this fusion module is beneficial but secondary in the model. Previous drug-+target interaction studies confirm that cross-attention outperforms simple fusion strategies such as concatenation and element-wise addition [5]. The results support this viewpoint. Most existing works regard cross-modal fusion as the core for interaction modeling. In the framework with LLM semantic pre-enhancement, this attention module only serves as an auxiliary unit for interaction representation learning.

#### 2.3.2. Experimental Validation of Length-Adaptive Weighting

The length-adaptive weighting is compared with a uniform weighting baseline. Uniform weight assigns a fixed value for the fusion of structural and semantic features of proteins and drug molecules. Length-adaptive weight assigns different values based on protein-length intervals. Experiments are conducted on the BindingDB and BioSNAP datasets, with results reported in Table 2 and Table 3, respectively.

On BindingDB, consistent gains are observed. The F1 score improves by 1.19%, followed by accuracy (+1.01%). These results demonstrate that protein-length-based weight allocation effectively enhances feature fusion. On BioSNAP, length-adaptive weighting achieves better performance across all metrics. Notable improvements include precision (+4.07%) and accuracy (+1.13%).

#### 2.3.3. Cold-Start Ablation Experiments

To further verify the importance of the enhanced LLM module, experiments are conducted on the Full model and the M2 model under the cold-start scenario of the BioSNAP dataset. AUROC, AUPRC, recall, F1 score and accuracy are adopted as evaluation metrics, and the results are presented in Figure 4b.

In particular, the Full model outperforms the M2 model by approximately 8 to 10 percentage points in AUROC and AUPRC, with the gap in accuracy reaching nearly 10 percentage points. This fully demonstrates that the enhanced LLM module can provide effective biomedical semantic information for the model, enabling it to maintain stable and reliable prediction performance when facing unknown drug–protein pairs.

Cold-start experiments show that BioLA-DTI can effectively predict proteins and drugs not present in the training set. In practice, many new targets (e.g., orphan receptors) and new compounds lack known interaction records [33]. However, their sequences, annotations, and molecular structures can be obtained from existing databases. Relying solely on this information for novel drug–target pair prediction has limited reliability [34]. To address this, BioLA-DTI combines textual protein features generated by BioGPT, protein sequence features, and structural drug features. This assists in predicting potential interactions for low-resource drug–target pairs. Compared with traditional methods, the model reduces dependence on known pairs in the training set. It is suitable for initial screening of new targets and new drugs, as well as drug repurposing for rare diseases.

### 2.4. Cold-Start Experiment

The performance comparison of all baseline methods under the cold-start scenario on the BindingDB dataset is shown in Figure 3a, with AUROC and AUPRC adopted as evaluation metrics. In the BindingDB cold-start setting, the proposed method significantly outperforms all comparison models in terms of AUROC, indicating that the model possesses an outstanding discriminative ability to distinguish interactive from non-interactive unknown drug–target pairs.

Meanwhile, it also achieves competitive AUPRC performance, which further verifies the model’s capability of accurately identifying positive samples.

The performance of all comparison methods in the cold-start scenario of the BioSNAP dataset is shown in Figure 3b. AUROC and AUPRC are used as evaluation metrics.

In the BioSNAP cold-start setting, the proposed method achieves relatively good performance in both AUROC and AUPRC, ranking among the top performers. It maintains stable and competitive discriminative performance and exhibits better robustness and adaptability. These results suggest that BioLA-DTI can serve as an effective preliminary screening tool for low-resource targets, reducing the cost of experimental validation in drug repurposing pipelines.

### 2.5. Visualization of Predicted Probability Distribution

**Figure 5:**
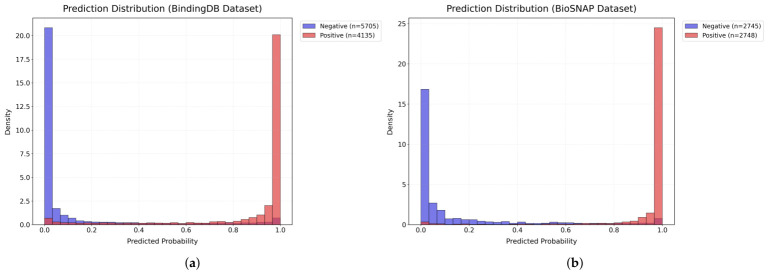
Prediction distribution for positive/negative samples. (**a**) Results on BindingDB. (**b**) Results on BioSNAP.

The predicted probability distributions of positive and negative samples of the proposed method on the BindingDB and BioSNAP test sets are shown in Figure 5a and Figure 5b, respectively. The y-axis shows probability density, normalized separately for each class (positive/negative), not over the entire dataset. Thus, each class histogram integrates to 1. This within-class normalization highlights distributional shapes despite class imbalance. Sample sizes are given in the legend. The predicted probabilities of negative samples are highly concentrated near 0, while those of positive samples cluster closely around 1. The two types of samples are almost completely separated in the probability space, with no obvious sample distribution in the transitional interval.

The results demonstrate that the DTI prediction model proposed in this work possesses an extremely strong ability to distinguish positive from negative samples on both the BindingDB and BioSNAP datasets. It achieves high confidence in prediction results, with a clear decision boundary and superior overall model performance.

## 3. Discussion

Predicting whether drug molecules can bind effectively with specific proteins is a core task in computer-aided drug design (CADD). In recent years, deep learning has made remarkable progress in this field due to its powerful feature extraction capability. TC-DTA [10], HyperAttentionDTI [35] and DeepPurpose [36] treat drug SMILES strings and protein amino acid sequences as 1D sequences, representing typical unimodal methods. They adopt 1D convolutional neural networks or recurrent neural networks for encoding. These methods extract local patterns and sequential dependencies to predict binding affinity. Sequence-based approaches have simple inputs and no need for complex structural computation. They are also easy to implement. Nevertheless, 1D sequences inevitably lose the spatial topology of drug molecules and the relative positions of functional groups. This is an intrinsic property of such representations because SMILES strings and amino acid sequences are inherently linear projections of three-dimensional molecular structures. They also have limited capacity to capture long-range dependencies among distant amino acid residues in proteins. Such dependencies are critical for protein folding and binding-site formation.

To compensate for the loss of structural information in unimodalmethods, researchers have introduced graph neural networks. Such methods have found extensive applications in both biomedical and logical reasoning tasks [37]. They model drug molecules as topological graphs while retaining protein-sequence representations. This marks an initial step toward multi-modal modeling. Specifically, MGraphDTA [11], GUNE-DTA [38] and GraphCPI [39] treat atoms as nodes and chemical bonds as edges. They adopt graph convolutional networks to extract spatial features, significantly enhancing the perception of stereochemical drug properties. However, these early bimodalmethods still adopt relatively simple fusion strategies. They typically concatenate drug graph features with protein-sequence features before feeding them into the prediction layer, lacking explicit modeling of deep interactions between the two modalities. Moreover, these methods still require the explicit construction of graph structures from protein sequences. The scale of such graphs grows quadratically with sequence length, leading to high computational complexity and limiting their applicability to long-chain proteins.

To overcome the limitation of simple concatenation in bimodalmethods, researchers have introduced attention mechanisms. These mechanisms enable more fine-grained cross-modal interaction. Methods such as DrugBAN [30], iNGNN-DTI [40] and MIFAM-DTI [41] retain drug graph features and protein-sequence features. They employ bidirectional attention or cross-attention modules. These modules explicitly model local interactions between drugs and proteins. Compared to simple concatenation approaches, these methods achieve superior performance on multiple public benchmarks. However, existing fusion strategies all follow a static fusion paradigm. This holds true for both early simple concatenation and fine-grained cross-attention. Under this paradigm, identical fusion parameters are used for all protein samples. The impact of protein sequence-length heterogeneity is not considered. In fact, short-chain proteins have concentrated functional sites. Their sequences already contain sufficient discriminative information. In contrast, long-chain proteins contain multiple complex structural domains. Their key functional sites are sparsely distributed. Thus, they require rich contextual knowledge. Static fusion cannot adapt to these two distinct scenarios [42]. It may introduce redundant noise for short proteins. It also suffers from information insufficiency for long proteins. Meanwhile, large language models (e.g., BioGPT and PubMedBERT) can generate protein descriptions rich in biomedical semantics, providing a new avenue for the supplementation of missing knowledge for long-chain proteins. However, existing work simply concatenates such semantic features with structural features, lacking a mechanism to dynamically adjust the fusion intensity of LLM knowledge according to protein length.

Existing methods are capable of extracting structural features and association information from drugs and proteins, yet they suffer from two notable deficiencies. Primarily, most models fail to account for the heterogeneity in protein sequence lengths. Moreover, these models encounter difficulties in adaptively integrating knowledge from large language models. Such limitations consequently impair the performance improvement afforded by multi-modal fusion in prediction tasks. To address the aforementioned common shortcomings, a length-adaptive large language model-enhanced framework for drug–target interaction (DTI) classification is proposed. Using the BioSNAP and BindingDB datasets, the proposed method is evaluated via quantitative comparisons, ablation studies, and probability distribution visualizations. All quantitative metrics consistently demonstrate the effectiveness of the designed dynamic fusion strategy. To validate the model’s positive predictions biologically, this study designs a visual case analysis based on molecular conformations. The next section selects two groups of drug–target pairs with high-confidence positive predictions as research cases for interpretable analysis via molecular docking visualization.

### Case Studies

**Figure 6:**
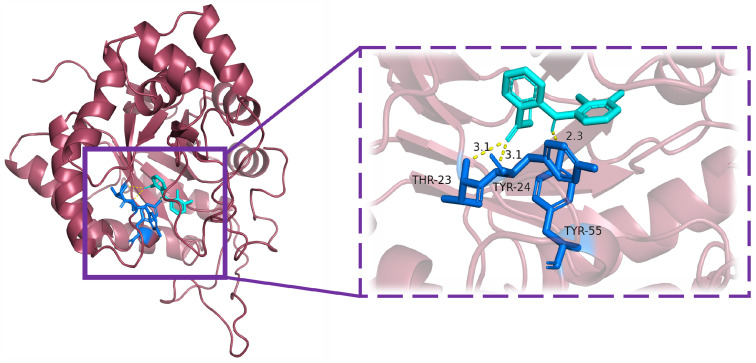
3D complex structure obtained by molecular docking of Mefenamic acid with Q04828.

**Figure 7:**
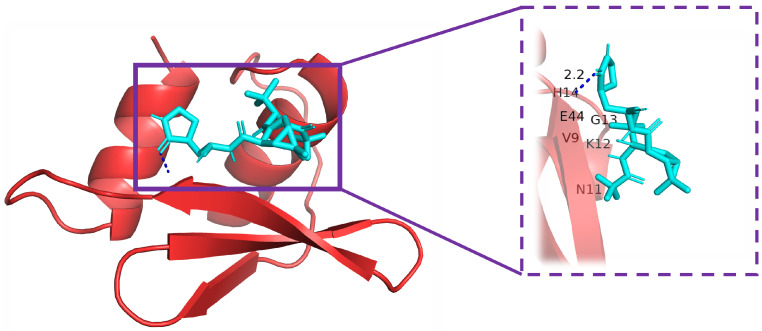
3D complex structure obtained by molecular docking of Nirmatrelvir with the main SARS-CoV-2 protease.

To further evaluate the interpretability and practical drug discovery value of BioLA-DTI, two drug–target pairs with high positive prediction confidence are selected for case analysis: mefenamic acid (DB00784) and its target, Q04828, as well as CID 155903259 and its target, UniProt ID P0DTD1 (Figure 6 and Figure 7). Among them, red represents the target protein, cyan represents the drug molecule, and blue indicates hydrogen bond interactions. In Figure 6, mefenamic acid forms multiple hydrogen bonds with several polar residues inside the pocket of target protein Q04828. In Figure 7, Nirmatrelvir establishes hydrogen-bond interactions with residues within the active region of the main SARS-CoV-2 protease. AutoDock Vina (1.5.7) is adopted to independently calculate the 3D binding conformations between small molecules and target proteins. Notably, the presented 3D binding diagrams serve only as visual aids to illustrate potential interaction patterns between ligands and target proteins.

The two high-confidence positive prediction cases, combined with the docking-visualized binding modes, demonstrate that BioLA-DTI can serve as an effective front-end filtering tool in virtual screening pipelines. It can rapidly screen out high-probability positive candidate molecules from large compound libraries. This significantly reduces the search space for subsequent computationally expensive molecular docking or molecular dynamics simulations. Ultimately, it improves the overall efficiency of the virtual drug screening workflow.

## 4. Materials and Methods

### 4.1. Datasets

Widely adopted public biomedical benchmark datasets are utilized for model validation to guarantee reliable experimental results and impartial evaluation. Detailed information about the datasets is presented as follows.

BioSNAP [43] is derived from the Stanford BioSNAP biomedical network and constructed based on the DrugBank database. It contains experimentally validated interaction pairs between approved drugs and target proteins. This dataset is fully balanced with a positive-to-negative sample ratio of 1:1. As listed in Table 4, it includes 27,464 interaction samples, covering 4510 drugs and 2181 proteins. Figure 1a,b show the density distributions of ligand SMILES lengths and protein sequence lengths in the BioSNAP dataset. In this dataset, drug SMILES strings exhibit a unimodal distribution, with most lengths concentrated between 20 and 100 characters. Protein sequences follow a long-tailed distribution, with most lengths ranging from 200 to 600 residues and a notable number exceeding 1000 residues. This significant variation in protein length further motivates the length-adaptive fusion module.

BindingDB [44] collects compound-target binding affinity data validated by literature and patents. This study uses its standard low-bias preprocessed version. The samples are labeled according to the binding affinity: samples with $K_{d}\leq$ 30 nM are defined as positive, and those with $K_{d}>30$ nM are defined as negative. The dataset is slightly imbalanced, with a positive–negative ratio of approximately 5:7. It contains 49,199 interaction samples involving 14,643 drugs and 2623 proteins, as listed in Table 4. Figure 2a,b show the density distributions of ligand SMILES lengths and protein sequence lengths in the BindingDB dataset. In this dataset, most drug SMILES strings range from 20 to 80 characters, while protein sequences exhibit a long-tailed distribution with large variations in length. This variation motivates our introduction of the length-adaptive fusion module.

### 4.2. Evaluation Metrics and Implementation

#### 4.2.1. Evaluation Metrics

This paper adopts multiple mainstream metrics to comprehensively evaluate the model’s drug–target interaction prediction performance. These metrics include AUROC, AUPRC, accuracy, precision, recall, and F1 score. Among them, AUROC measures the model’s overall ability to distinguish between positive and negative samples across different thresholds. Values closer to 1 indicate stronger discriminative ability. AUPRC reflects the trade-off between precision and recall and is more sensitive to the identification performance of the minority class in imbalanced data scenarios. Accuracy is the proportion of correctly predicted samples among all samples, reflecting the overall correctness of the model’s predictions. Precision is the proportion of true-positive samples among all samples predicted as positive, measuring the exactness of the predictions. Recall is the proportion of actual positive samples correctly identified by the model, measuring the completeness of the model in capturing positive samples. The F1-score is the harmonic mean of precision and recall, providing a comprehensive reflection of both metrics, and is commonly used for holistic evaluation in imbalanced data scenarios [31,45]. The definitions of each metric are presented as follows.

(1) $$\begin{array}{cc} \mathrm{AUROC} & =\int_{0}^{1}\mathrm{TPR}\left(k\right)\;d\left(\mathrm{FPR}\left(k\right)\right) \end{array}$$

(2) $$\begin{array}{cc} \mathrm{AUPRC} & =\int_{0}^{1}\mathrm{Precision}\left(k\right)\;d\left(\mathrm{Recall}\left(k\right)\right) \end{array}$$

(3) $$\begin{array}{cc} \mathrm{Accuracy} & =\frac{TP+TN}{TP+TN+FP+FN} \end{array}$$

(4) $$\begin{array}{cc} \mathrm{Precision} & =\frac{TP}{TP+FP} \end{array}$$

(5) $$\begin{array}{cc} \mathrm{Recall} & =\frac{TP}{TP+FN} \end{array}$$

(6) $$\begin{array}{cc} F1\text{-}\mathrm{score} & =2\times \frac{\mathrm{Precision}\times \mathrm{Recall}}{\mathrm{Precision}+\mathrm{Recall}} \end{array}$$

Specifically, TP (True Positives) is the number of true-positive samples. TN (True Negatives) is the number of true-negative samples. FP (False Positives) is the number of false-positive samples, and FN (False Negatives) is the number of false-negative samples. All metrics range from 0 to 1, and higher values indicate better model performance.

#### 4.2.2. Implementation

To ensure experimental reproducibility and the reliability of results, standardized experimental parameters and training strategies are adopted in this work. The detailed settings are presented as follows:

- Training setup: 5-fold cross-validation is employed with a maximum of 100 training epochs. Early stopping is adopted with patience set to 15, monitoring the AUROC on the validation set. The weight decay is set to 1 × 10^−4^, and the ReduceLROnPlateau learning rate scheduler is used with a decay factor of 0.5 and patience of 10.
- LLM configuration: BioGPT (microsoft/BioGPT) is deployed locally. PubMedBERT is used to encode descriptive text, and the projection layers are set to be trainable.
- Cold-start setting: An independent test set is strictly constructed with no overlapping drugs or proteins between the training and test sets. The test set contains drugs and proteins that are completely unseen during training. This rigorous setting enables a fair evaluation of the model’s generalization ability for unknown drugs and proteins. AUROC and AUPRC are adopted as evaluation metrics.

### 4.3. Method

**Figure 8:**
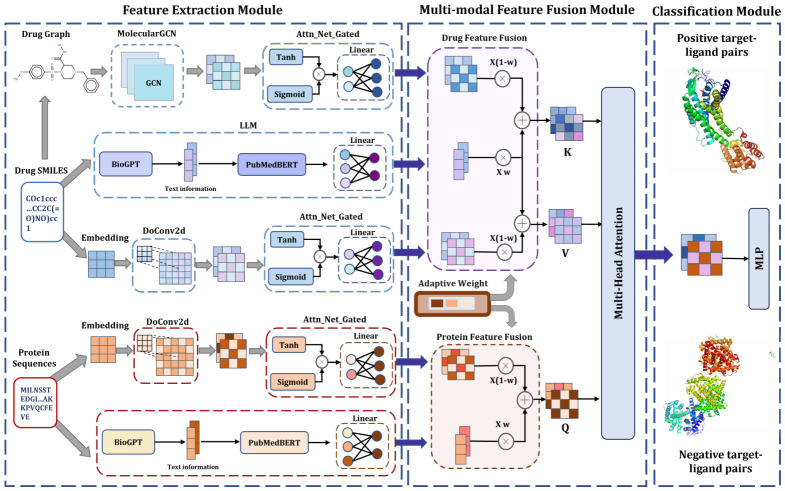
Overall architecture of BioLA-DTI, consisting of feature extraction, length-adaptive fusion, and classification modules. Drug and protein features are extracted via sequence encoders, GCN (Graph Convolutional Network), and biomedical LLMs, then dynamically weighted according to protein sequence length, adaptively fused, and fed into an attention-enhanced MLP for interaction prediction.

**Figure 9:**
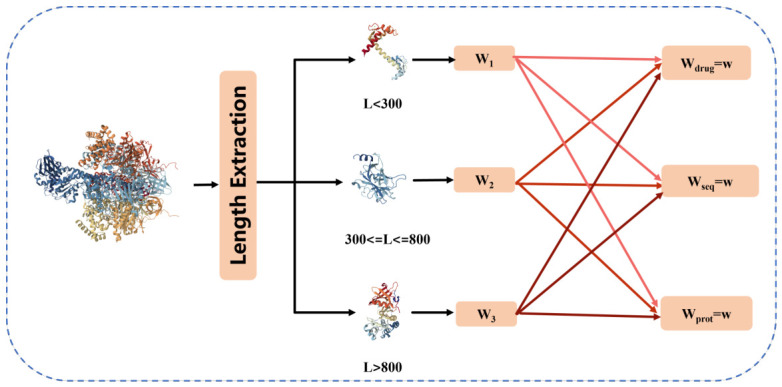
Weight allocation of LLM-derived features based on 300/800 residue boundaries (BindingDB & BioSNAP Distribution-Guided).

The overall framework of the proposed method is shown in Figure 8. It consists of a feature extraction module, a multi-modal feature fusion module and a classification module. The feature extraction module constructs multi-modal and multi-level representations for drugs and proteins. It includes three complementary perspectives: molecular graph, sequence, and LLM semantic information. The multi-modal feature fusion module adopts a length-adaptive weight mechanism. It achieves adaptive fusion of features for individual entities. Meanwhile, it employs multi-head attention to model deep interactions between drug and protein features. The adaptive weight mechanism is illustrated in Figure 9. The classification module takes the fused interactive features as input. It uses a multi-layer perceptron to finally predict the probability of drug–target interaction.

#### 4.3.1. Feature Extraction Module

Step 1: Sequence Feature Extraction

The protein sequence is first integer-encoded and rearranged. It is flattened by DO-Conv2d to obtain the sequence feature.

Let P denote the input feature map with $C_{\mathrm{in}}$ input channels and $C_{\mathrm{out}}$ output channels. Let $K_{h}\times K_{w}$ be the kernel size and $D_{\mathrm{mul}}$ be the depth multiplier for over-parameterization. DO-Conv2d maintains two independent learnable kernels in training:

- Pointwise kernel: $W\in R^{C_{\mathrm{out}}\times D_{\mathrm{mul}}\times C_{\mathrm{in}}}$;
- Depthwise kernel: $D\in R^{C_{\mathrm{in}}\times \left(K_{h}\cdot K_{w}\right)\times D_{\mathrm{mul}}}$.

In forward propagation, the two kernels are fused into a unified 4D composite kernel ($W^{\prime }\in R^{C_{\mathrm{out}}\times C_{\mathrm{in}}\times K_{h}\times K_{w}}$) via tensor contraction. The fusion formulation is written as

(7) $$W_{c_{\mathrm{out}},c_{\mathrm{in}},i,j}^{\prime }=\sum_{d=1}^{D_{\mathrm{mul}}}W_{c_{\mathrm{out}},d,c_{\mathrm{in}}}\cdot D_{c_{\mathrm{in}},\left(i-1\right)K_{w}+j,d},$$

where $i\in [1,K_{h}]$ and $j\in [1,K_{w}]$ index spatial positions inside the kernel. Standard 2D convolution is then conducted with the fused kernel on input feature map P:

(8) $$O=W^{\prime }*P.$$

Following original DO-Conv settings, D adopts fixed diagonal initialization with $d_{\mathrm{diag}}$. The initialized composite kernel approximates a vanilla convolution kernel. Stable convergence is guaranteed at early training epochs.

After sequence feature extraction, a gating module is introduced to refine representations. Convolution layers generate gate values, followed by Sigmoid normalization. The mathematical formulation is given as follows:

(9) $$H_{p}^{\mathrm{gate}}=H_{p}^{\mathrm{raw}}⊙g$$

Then, features are obtain via global average pooling ($h_{d}^{\mathrm{struct}}\in R^{128}$).

The drug SMILES string is embedded first. DO-Conv2d is used to capture local sequence features. The feature map is then flattened and processed by a linear layer ($h_{d}^{\mathrm{seq}}$). The gating mechanism follows the same principle as that for proteins.

Step 2: Molecular Graph Feature Extraction

The drug SMILES is first converted into a DGL graph with initial atom features. Node features are obtained through three GCN layers. Global average pooling is then applied to obtain $h_{d}^{\mathrm{graph}}\in R^{128}$.

Step 3: Semantic Feature Extraction

We introduce BioGPT to construct prompts for drug SMILES and protein sequences (e.g., “Describe the pharmacological properties of this molecule”). The generation temperature is set to 0.6, with a maximum of 120 new tokens, and the results are cached.

During encoding, PubMedBERT extracts the token vector, which is then projected via two linear layers to obtain $f_{d}^{\mathrm{llm}}\in R^{128}$.

#### 4.3.2. Multi-Modal Feature Fusion Module

Step 1: Length-Adaptive Fusion

For proteins of varying lengths, we design a length-adaptive weighted fusion mechanism based on the actual sequence length. It dynamically adjusts the weights of original features and LLM pre-trained features to achieve balanced fusion. First, the input protein sequence is preprocessed by removing padding tokens, and the true amino acid number is counted as the actual length (*L*). Then, protein sequences are divided into short, medium and long groups according to length. Independent weights ($w_{1}$, $w_{2}$, and $w_{3}$) are defined for each group.

The actual protein length (*L*, after padding removal) is defined as follows:

(10) $$\alpha \left(L\right)=\left\{\begin{array}{cc} w_{1}, & L<300\\ w_{2}, & 300\leq L\leq 800\\ w_{3}, & L>800 \end{array}\right.$$

Based on the above adaptive weight (α), weighted fusion is performed on three types of features and pretrained LLM features to realize complementary enhancement of multi-modal information.

(11) $${\tilde{h}}_{d}^{\mathrm{seq}}=\left(1-\alpha \right)h_{d}^{\mathrm{seq}}+\alpha f_{d}^{\mathrm{llm}}$$

(12) $${\tilde{h}}_{d}^{\mathrm{graph}}=\left(1-\alpha \right)h_{d}^{\mathrm{graph}}+\alpha f_{d}^{\mathrm{llm}}$$

(13) $${\tilde{h}}_{p}=\left(1-\alpha \right)h_{p}^{\mathrm{struct}}+\alpha f_{p}^{\mathrm{llm}}$$

The fused vector is expanded to a sequence length of 1 for attention computation. This design adaptively adjusts the contribution of LLM features based on protein length, reducing their influence for short proteins and enhancing it for long-chain proteins.

Step 2: Learnable Multi-Head Attention Fusion

The query, key and value vectors of the multi-head attention module are constructed from length-adaptive LLM-weighted multi-modal fused features.

- Query Vector (*Q*): The fused protein feature (${\tilde{h}}_{p}$) serves as query vector (*Q*). It is calculated by adaptively weighting raw protein convolutional features and protein LLM features with a learnable coefficient (α). The model uses proteins as query carriers to extract interaction signals from multi-modal drug features.
- Key Vector (*K*): The fused drug graph feature (${\tilde{h}}_{d_{\mathrm{graph}}}$) acts as key vector (*K*). It adaptively fuses GCN-extracted topological drug features and drug LLM embeddings to encode 3D molecular structures and establish topological matching relationships with proteins.
- Value Vector (*V*): The fused drug SMILES sequence feature (${\tilde{h}}_{d_{\mathrm{seq}}}$) functions as value vector (*V*). It combines 1D convolution drug sequence features and drug LLM features via weighted aggregation. This feature also works as matching keys for the sequence branch and retains semantic information of linear molecular strings.

The model adopts the three groups of *Q*, *K*, and *V* vectors. It produces three separate attention maps to reveal correlations between proteins and multi-modal drug features. Details are listed as follows:

- Drug graph–protein attention ($A_{1}$) characterizes the correlation between protein and drug molecular structural features;
- Drug sequence–protein attention ($A_{2}$) characterizes the correlation between protein and drug SMILES sequence features;
- Fused drug–protein attention ($A_{3}$) characterizes the correlation between protein and the fused features of the drug graph and sequence.

*d* denotes the feature dimension of single-head attention. A learnable weight vector ($\alpha =[\alpha_{1},\alpha_{2},\alpha_{3}]$) is introduced, whose weights ($\beta_{1}$, $\beta_{2}$, and $\beta_{3}$) are obtained by Softmax normalization (ensuring their sum is 1) to adaptively balance the contributions of the three attention maps. The final fused attention map (*A*) is the weighted sum of the three attention maps:

(14) $$A_{1}=\mathrm{Softmax}\left(\frac{KQ^{⊤}}{\sqrt{d_{k}}}\right)$$

(15) $$A_{2}=\mathrm{Softmax}\left(\frac{VQ^{⊤}}{\sqrt{d_{k}}}\right)$$

(16) $$\begin{array}{cc} A_{3} & =\mathrm{Softmax}\left(\frac{\left(V+K\right)Q^{⊤}}{\sqrt{d_{k}}}\right) \end{array}$$

(17) $$\left[{\tilde{\beta }}_{1},{\tilde{\beta }}_{2},{\tilde{\beta }}_{3}\right]=\mathrm{Softmax}\left([\alpha_{1},\alpha_{2},\alpha_{3}]\right)$$

(18) $$A={\tilde{\beta }}_{1}A_{1}+{\tilde{\beta }}_{2}A_{2}+{\tilde{\beta }}_{3}A_{3}$$

This design takes proteins as queries to retrieve complementary cues from sequential and structural drug modalities. It faithfully models the induced-fit effect underlying drug–target interactions. The trainable weight mechanism enables adaptive integration of heterogeneous multi-modal features. It yields biologically meaningful embeddings for subsequent binary prediction tasks.

#### 4.3.3. Loss Function

In DTI prediction, some positive samples share high feature similarity. These samples are easily misclassified. Traditional cross-entropy loss hardly guides the model to focus on such hard samples. PolyLoss [46] extends cross-entropy loss by a polynomial expansion term. It imposes additional penalties on positive samples with large prediction errors. This forces the model to pay more attention to boundary samples. PolyLoss is employed as the optimization objective function to strengthen the model’s discrimination performance on hard samples.

(19) $$L_{\mathrm{CE}}=-1/N\sum_{i}\left[y_{i}\log\left({\hat{y}}_{i}\right)+\left(1-y_{i}\right)\log\left(1-{\hat{y}}_{i}\right)\right]$$

(20) $$L_{\mathrm{Poly}}=L_{\mathrm{CE}}+\delta \cdot 1/N\sum_{i}\left(1-{\hat{y}}_{i}\right)$$

where $y_{i}$ denotes the true label of the *i*-th sample, *N* represents the number of samples in a batch, and δ is a hyperparameter that controls the strength of the polynomial correction term. The first term ($L_{\mathrm{CE}}$) is the standard cross-entropy loss. The second term ($\delta \cdot 1/N\sum_{i}\left(1-{\hat{y}}_{i}\right)$) imposes an additional penalty on the prediction error of positive samples. By doing so, this loss alleviates the common class-imbalance issue in DTI tasks and improves the model’s recognition performance on minority classes. Whereas Focal Loss [47] relies on exponential decay to reduce the loss from easy samples, PolyLoss controls only the first-order term weight in the loss expansion with a single lightweight hyperparameter. This achieves explicit regularization on hard positive samples without incurring the computational overhead of complex exponential operations. Subsequently, the asymmetric polynomial loss (APL) variant [48] further validated the effectiveness of polynomial-type loss designs in mitigating positive–negative sample imbalance in relation prediction tasks. Concurrently, a DTI-specific study published in the *Briefings in Bioinformatics* journal confirmed that PolyLoss can effectively address the issue of feature homogenization among interacting samples [29]. These findings collectively substantiate the adaptability and extensibility of this loss paradigm in bioinformatics interaction prediction scenarios.

## 5. Conclusions

This paper proposes a length-adaptive, LLM-enhanced method named BioLA-DTI for drug–target interaction prediction. Its core idea is to adjust the fusion weights between LLM-derived semantic features and conventional structural features according to protein sequence length. This addresses the problems of noise accumulation in short proteins and under-representation in long proteins. Extensive experiments on BioSNAP and BindingDB show that BioLA-DTI consistently outperforms state-of-the-art baseline models across multiple evaluation metrics. It performs particularly well under cold-start settings. Ablation experiments confirm that LLM semantic enhancement, the gating mechanism, and learnable multi-head attention fusion all contribute significantly to performance improvement. The above results show that the model can generate targeted candidate molecule lists for scarce targets. This supports drug repurposing studies. Unlike existing models that rely on a single modality, BioLA-DTI has a length-adaptive perception capability. This gives it a unique generalization advantage when predicting targets with extreme sequence lengths (e.g., short peptides or long, disordered proteins). It effectively overcomes the limitations of conventional methods, which accumulate noise on short sequences and lack adequate representation for long ones.

In terms of application, the framework can be directly used for virtual screening of orphan receptors with low sequence homology. This reduces the experimental costs of blind high-throughput screening. In addition, BioLA-DTI can serve as a front-end pre-filtering tool in large-scale virtual screening pipelines. The model enables rapid preliminary screening to select a subset of compounds with high potential binding activity from massive compound libraries. This substantially decreases the computational cost of subsequent high-precision molecular docking and molecular dynamics simulations. Ultimately, it improves the overall efficiency of the entire virtual drug screening workflow.

It should be noted that the length thresholds (300 and 800) used in the method are based on common protein length distributions. They may not be optimal for certain special families, such as very short antimicrobial peptides or very long cytoskeletal proteins [49]. Future work will explore more refined weight functions based on domain number or intrinsically disordered region proportions. The method will also be applied to practical drug repositioning projects, especially for rapid screening of orphan receptors and emerging pathogen targets.

## Figures and Tables

**Table 1:** Performance comparison of various methods on the BioSNAP and BindingDB datasets.

Dataset	Method	AUROC	AUPRC	Recall	F1 Score	Accuracy
BindingDB	DeepConv-DTI	0.945 ± 0.002	0.925 ± 0.005	0.873 ± 0.006	0.884 ± 0.018	0.882 ± 0.009
	MolTrans	0.952 ± 0.002	0.936 ± 0.001	0.877 ± 0.006	0.886 ± 0.016	0.887 ± 0.009
	TransformerCPI	0.941 ± 0.003	0.925 ± 0.005	0.876 ± 0.007	0.854 ± 0.008	0.875 ± 0.006
	GraphDTI	0.951 ± 0.002	0.934 ± 0.003	0.882 ± 0.012	0.888 ± 0.003	0.888 ± 0.005
	MCANet	0.958 ± 0.001	0.827 ± 0.003	0.889 ± 0.011	0.884 ± 0.002	0.903 ± 0.003
	DrugBAN	0.956 ± 0.001	0.943 ± 0.002	0.892 ± 0.003	0.901 ± 0.004	0.897 ± 0.002
	MGNDTI	0.958 ± 0.002	0.943 ± 0.003	0.899 ± 0.003	0.902 ± 0.004	0.899 ± 0.004
	MGMA-DTI	0.963 ± 0.003	**0.951 ± 0.005**	0.913 ± 0.011	0.898 ± 0.004	**0.914 ± 0.006**
	This work	**0.964 ± 0.001**	0.948 ± 0.002	**0.915 ± 0.003**	**0.910 ± 0.002**	0.907 ± 0.001
BioSNAP	DeepConv-DTI	0.886 ± 0.006	0.891 ± 0.006	0.761 ± 0.029	0.782 ± 0.013	0.805 ± 0.009
	MolTrans	0.887 ± 0.004	0.891 ± 0.005	0.755 ± 0.031	0.764 ± 0.023	0.802 ± 0.011
	TransformerCPI	0.897 ± 0.006	0.903 ± 0.009	0.793 ± 0.003	0.802 ± 0.007	0.821 ± 0.007
	GraphDTI	0.891 ± 0.003	0.898 ± 0.004	0.767 ± 0.010	0.802 ± 0.006	0.811 ± 0.006
	MCANet	0.903 ± 0.006	0.773 ± 0.005	0.839 ± 0.006	0.832 ± 0.004	0.829 ± 0.003
	DrugBAN	0.896 ± 0.005	0.901 ± 0.011	0.828 ± 0.011	0.834 ± 0.003	0.834 ± 0.004
	MGNDTI	0.905 ± 0.003	0.908 ± 0.004	0.852 ± 0.007	0.833 ± 0.005	0.829 ± 0.006
	MGMA-DTI	0.909 ± 0.011	0.911 ± 0.008	0.858 ± 0.006	**0.857 ± 0.008**	0.845 ± 0.003
	This work	**0.924 ± 0.002**	**0.929 ± 0.003**	**0.876 ± 0.014**	0.853 ± 0.004	**0.846 ± 0.004**

**Table 2:** Ablation study of length-adaptive weighting on BindingDB.

Metrics	Length-Adaptive	Uniform	Improvement (%)
AUROC	0.9649	0.9594	+0.55%
AUPRC	0.9500	0.9446	+0.54%
F1-Score	0.9085	0.8966	+1.19%
Accuracy	0.9051	0.8950	+1.01%
Precision	0.8682	0.8626	+0.56%

**Table 3:** Ablation study of length-adaptive weighting on BioSNAP.

Metrics	Length-Adaptive	Uniform	Improvement (%)
AUROC	0.9272	0.9251	+0.21%
AUPRC	0.9331	0.9313	+0.18%
F1-Score	0.8548	0.8547	+0.01%
Accuracy	0.8518	0.8405	+1.13%
Precision	0.8471	0.8064	+4.07%

**Table 4:** Statistics of datasets.

Dataset	Drug	Protein	Interaction	Positive	Negative
BindingDB	14,643	2623	49,199	20,674	28,528
BioSNAP	4510	2181	27,464	13,830	13,634

## Data Availability

The datasets supporting the findings of this study are publicly available on GitHub at the following link: https://github.com/liLi0000L/BioLA-DTI.git (accessed on 26 May 2026).
